# Reproductive Outcomes of *In Vitro* Fertilization and Fresh Embryo Transfer in Infertile Women With Adenomyosis: A Retrospective Cohort Study

**DOI:** 10.3389/fendo.2022.865358

**Published:** 2022-07-29

**Authors:** Tingting Liang, Wen Zhang, Ningning Pan, Bing Han, Rong Li, Caihong Ma

**Affiliations:** ^1^ Center for Reproductive Medicine, Department of Obstetrics and Gynecology, Peking University Third Hospital, Beijing, China; ^2^ Department of Obstetrics and Gynecology, Second Hospital of Shanxi Medical University, Shanxi, China; ^3^ National Clinical Research Center for Obstetrics and Gynecology, Peking University Third Hospital, Beijing, China; ^4^ Key Laboratory of Assisted Reproduction, Ministry of Education, Peking University, Beijing, China; ^5^ Beijing Key Laboratory of Reproductive Endocrinology and Assisted Reproductive Technology, Beijing, China; ^6^ Research Units of Comprehensive Diagnosis and Treatment of Oocyte Maturation Arrest, Chinese Academy of Medical Sciences, Beijing, China

**Keywords:** adenomyosis, *in vitro* fertilization-embryo transfer, miscarriage rate, live birth rate, obstetric complications

## Abstract

**Background:**

Adenomyosis is commonly encountered in infertile women; however, it is still unclear whether adenomyosis has a detrimental effect on *in vitro* fertilization and embryo transfer (IVF-ET) outcomes.

**Method:**

We enrolled 1146 patients with adenomyosis and 1146 frequency-matched control women in a 1:1 ratio based on age, BMI, and basal follicle-stimulating hormone (FSH) level. After controlling for other factors, the rates of clinical pregnancy, miscarriage, live birth, and obstetric complications were compared between two groups.

**Results:**

There was no significant difference in clinical pregnancy rate between the two groups (38.1% vs. 41.6%; P=0.088). The implantation rate (25.6% versus 28.6%, P=0.027) and live birth rate (26% versus 31.5%, P=0.004) were significantly lower in the women with adenomyosis than in the controls. The miscarriage rate in the adenomyosis group was higher than that in the control group (29.1% versus 17.2%, P=0.001). After adjusting for confounding factors, multivariate analysis showed the clinical pregnancy rate was not statistically different between the two groups (OR: 0.852, P=0.070). In the adenomyosis group, the rate of miscarriage(OR: 1.877, P=0.000), placenta previa (OR: 2.996, P=0.042)and preeclampsia (OR: 2.287, P=0.042)were increased significantly, while live birth rate (OR: 0.541, P=0.000) was reduced significantly than control group.

**Conclusion:**

Adenomyosis has negative effect on IVF-ET outcomes in which miscarriage risk increased, live birth rate reduced and obstetric complications increased.

## Introduction

Adenomyosis is a benign gynaecological disease characterized by the presence of endometrial secretions and stroma in the myometrium as well as hyperplastic and hypertrophic muscular tissue, exacerbating chronic pelvic pain, dysmenorrhea, abnormal uterine bleeding, and subfertility in women of reproductive age (1). Due to enhanced ultrasound screening procedures and the advancing age of women seeking fertility treatment, adenomyosis in infertile women has become more common in recent years. The frequency of adenomyosis has been reported to be between 7% and 27% among infertile women (2).

Although the exact cause of adenomyosis and infertility is unclear, there is growing evidence that adenomyosis can adversely affect the outcome of *in vitro* fertilization and embryo transfer (IVF-ET) (3–5). Recently, through a systematic review and meta-analysis, Nirgianakis et al. discovered that adenomyosis was associated with a reduced clinical pregnancy rate and a high risk of miscarriage after assisted reproductive technology (ART) treatment (6). The studies included in this meta-analysis indicated that adenomyosis was associated with adverse pregnancy and neonatal outcomes irrespective of the mode of conception, but no analysis of obstetric complications was performed.

The heterogeneity identified in this meta-analysis was unavoidable and may be explained by differences in the study design, study power, criteria and instruments used to diagnose adenomyosis and in the selection of controls. According to Benaglia et al. (7), asymptomatic adenomyosis does not interfere with embryo implantation in women diagnosed with adenomyosis by transvaginal ultrasonography (TVS). A recent prospective study demonstrated that there was no difference in ART outcomes among women with adenomyosis and controls (8). However, the sample size in this study was small, with only 301 subjects. Additionally, although there is currently a lack of uniform criteria for ultrasound diagnosis of adenomyosis, the authors’ diagnostic criteria for adenomyosis involved only one feature, and the inclusion criteria were not strict. Therefore, the results of the study must be confirmed by further research involving a larger sample size.

Because of the limited number of observational studies and their heterogeneous design, the influence of adenomyosis on IVF/intracytoplasmic sperm injection (ICSI) prognosis is still unknown. It is well known that adenomyosis frequently coexists with other gynaecological disorders, such as endometriosis and uterine fibroids, and that this coexistence is linked to poor pregnancy outcomes (8, 9). Therefore, the influence of the confounding factors mentioned above on pregnancy outcomes must be excluded. In the present retrospective study, more than 1000 women with adenomyosis who underwent IVF/ICSI were enrolled, excluding those with decreased ovarian reserve and coexistence of severe endometriosis and fibroids, to determine whether adenomyosis has an impact on IVF treatment.

## Materials and Methods

At Peking University Third Hospital’s Reproductive Centre, a retrospective cohort study was conducted. We examined the medical records of all women who had their first cycle of IVF/ICSI treatment between January 2011 and December 2020. The patients were identified using the hospital’s online record system.

The following were the inclusion criteria: 1) patients with adenomyosis aged 40 years or less at the time of commencement of IVF/ICSI treatment; 2) patients diagnosed by at least two experienced sonographers and showing a uterine volume of ≥ 56 cm^3^ [the study of Sheth SS showed that the normal uterine volume range was 15-56 cm³ (10); the uterus volume was calculated using the formula long diameter x width diameter x anteroposterior diameter xπ/6 (11)] and two or more TVS-specific diagnostic criteria for adenomyosis (12); and 3) patients with freshly stimulated and day 3 cleavage-stage transfer cycles and tubal factor infertility screened by hysterosalpingography or laparoscopy. The following were the exclusion criteria: 1) laparoscopy-confirmed or TVS proposed endometriosis; 2) hydrosalpinx; 3) congenital uterine malformation or other uterine disorders (malformations, endometrial lesions, uterine fibroids distorting the uterine cavity); and 4) chromosomal anomaly (male or female partner). The control group women had a normal uterus and received IVF treatment but otherwise met the same selection criteria. The controls were chosen at random from the same database and matched 1:1 in terms of age, BMI, and basal follicle-stimulating hormone (FSH) level.

TVS diagnostic criteria for adenomyosis were exhibited the following: 1) an enlarged globular uterine configuration, 2) asymmetrical thickening of the myometrium, 3) absence of a junctional zone, 4) presence of heterogeneous endometrium areas, and 5) subendometrial striations and cysts (13, 14).

### ART Treatment Protocols

Controlled ovarian stimulation (COS) was performed on all participants by using recombinant or human menopausal gonadotrophins. When there were at least one to three follicles larger than 18 mm, a human chorionic gonadotrophin (hCG) button was administered 34 to 36 hours after hCG administration, and oocyte collection was performed with the help of ultrasound. Depending on the quality of the sperm, oocytes were inoculated using either conventional IVF or ICSI. The presence of two pronuclei (2PN) and two polar bodies (PBs) 17–19 hours after impregnation was used to determine fertilization. The Istanbul Consensus Workshop on Embryo Assessment criteria were used to evaluate embryo quality 68–72 hours (day 3) after insemination (15). Day 3 cleavage-stage embryos were either transferred or cultured for 48 hours to the blastocyst stage (16). Day 3 embryos were transferred with vaginal and/or intramuscular progesterone luteal support. ETs were carried out with the aid of a soft catheter (K-Soft 5100; Cook, Queensland, Australia).

Serum hCG levels were measured two weeks after transfer and were considered positive if they were greater than 10 IU. If an intrauterine gestational sac was visible, TVS at 30 days after transfer confirmed clinical pregnancy. Miscarriage was measured by the length of a clinical pregnancy before 24 weeks of gestation (early miscarriage was defined as foetal delivery before 12 weeks). Preterm delivery was defined as fetal delivery between 24 and 37 weeks of gestation, and severe preterm delivery was defined as fetal delivery before 32 weeks of gestation. Live birth was classified as a pregnancy that resulted in the birth of at least one living child, regardless of gestational age.

### Study Outcomes

The primary outcome was live birth rate per transfer cycle (number of live births divided by the number of women who received a transfer).

The secondary outcomes were as follows: 1) implantation rate (the number of gestational sacs divided by the number of transferred embryos); 2) clinical pregnancy rate (number of clinical pregnancies divided by the number of women who received a transfer); 3) miscarriage rate (the number of miscarriage pregnancies divided by the number of clinical pregnancies); 4) preterm delivery rate (number of preterm deliveries divided by the number of women who had delivery); and 5) pregnancy complications (including preeclampsia, gestational diabetes, placenta praevia and low birth weight (identified as a birthweight <2500 g).

### Statistical Analysis

The data were statistically analysed using SPSS version 25.0. (SPSS, Inc., Chicago, IL). The categorical variables were assessed with the chi-square test or Fisher’s exact test as appropriate. The means ± standard deviations (SDs) of the normally distributed quantitative variables were calculated and analysed using Student’s t test. The Mann–Whitney U test was used to analyse nonnormally distributed quantitative variables, which were expressed as medians (interquartile ranges). Logistic regression models were used to estimate the effect of adenomyosis on reproductive outcomes and obstetric complications. A probability (p) value of 0.05 was used to determine statistical significance (two-sided).

## Results

According to the standards described above, 2292 women were included in the study. The study group consisted of 1146 adenomyosis patients and 1146 women who served as controls. **Table 1** shows the demographics and benchmark characteristics of the entire study population. There were no significant differences between the groups in terms of age, BMI, baseline FSH level, or duration of infertility.

**Table 1:** Baseline characteristics of the study population.

Parameters	Adenomyosis (n= 1146)	Controls (n = 1146)	P value
Maternal age (year)	34.82±3.905	34.82±3.905	1
Infertility duration (year)	4 (2,7)	4 (2,7)	0.217
BMI (kg/m2)	23.18±3.02	23.16±3.03	0.991
AMH(ng/L)	2 (1,3)	2 (1,3)	0.050
Basal FSH (mIU/L)	5.85±3.01	5.84±3.05	0.543
Primary infertility	465/1146	485/1146	0.396

Normality quantitative variables, means ± SD.

Nonnormally distributed quantitative, median (IQR).

**Table 2** displays the features of the ART cycles and the embryology outcomes. The total dose of gonadotropin used and duration of ovarian stimulation were significantly higher in the women with adenomyosis than in the controls. There was no difference in the number of women assigned to a certain number of ICSI cycles (29.84% vs. 27.4%; P=0.212). There were no significant differences between the two groups in terms of ovarian response to simulation, number of oocytes retrieved, fertilization rate, endometrial thickness on the hCG trigger day, or number of embryos transferred.

**Table 2:** Characteristics of IVF-ICSI stimulation cycles.

Parameters	Adenomyosis (n = 1146)	Controls (n = 1146)	P value
Ultra-long GnRHa protocol, % (n)	52.2 (599/1146);	14.5 (167/1146);	<0.001
Long GnRH-agonist, % (n)	19.4 (223/1146);	33.4(383/1146)	<0.001
GnRH-antagonist protocols, % (n)	23.8 (273/1146);	45.7 (524/1146);	<0.001
Short GnRH-agonist, % (n)	4.4 (51/1146)	6.2 (72/1146)	0.052
Dose of gonadotrophins (IU)	3450 (2550,5471);	2850 (2138,3784);	<0.001
Length of ovarian stimulation (days)	11.65±2.52	11.22±2.41	<0.001
EM thickness on HCG-day (mm)	10.73±1.90	10.66±1.74	0.373
E2 on hCG-day (pmol/L)	5774 (3480, 9765)	5531 (3788,8062)	0.109
P on hCG-day (nmol/L)	1.91 (1.31,2.59)	1.90 (1.43,2.67)	0.523
Number of oocytes collected	10 (6,13)	10 (5,12)	0.621
IVF,n(%)	70.16 (804/1146)	72.51 (831/1146)	0.212
ICSI, n (%)	29.84 (342/1146)	27.49 (315/1146)	0.212
2PN embryos	6 (1,8)	6 (3,8)	0.634
Available embryos	4 (2,5)	4 (2,5)	0.747
Number of embryos transferred	1.87±0.49	1.84±0.42	0.116

Normality quantitative variables:means ± SD.

Nonnormally distributed quantitative:median (IQR).

E2, estradiol; EM, endometrial; hCG, human chorionic gonadotrophin; ICSI, intracytoplasmic sperm injection; IVF, in vitro fertilization; 2PN, two pronuclear zygote; P, progesterone.

**Table 3** contains the actual parameters during IVF/ICSI treatment as well as the clinical outcomes of the adenomyosis and tubal cohorts. The embryo implantation rate was significantly different between the two groups (25.6% vs. 28.6%; P=0.027). The difference in clinical pregnancy between the two groups was not statistically significant (38.1% vs. 41.6%; P=0.088). The miscarriage rate in the study group was significantly higher than that in the control group (29.1% vs. 17.2%; P=0.001). The live birth rate was 31.5% in the control group and 26% in the study group (P=0.004), and there was no difference in the preterm birth rate between the two groups (20.1% vs. 16.6%; P=0.245); however, the incidence of severe preterm delivery was significantly higher in the adenomyosis group (7.72% vs. 1.66%; P<0.001). The incidence of placenta previa, preeclampsia and low birth weight was significantly higher in the adenomyosis group than in the control group (4% vs. 1.4%, 6% vs. 2.8% and 21.9% vs. 10.8%, respectively, P=0.033, P=0.038, P<0.001). The difference in average birth weight between the adenomyosis and control groups was significant (P=0.014).

**Table 3:** Reproductive outcomes calculated per fresh ET cycle.

Variable	adenomyosis	controls	OR (95% CI)	P value
Implantation rate, n (%)	25.6 (550/2147)	28.6 (597/2084)	0.858 (0.749-0.982)	0.027
Clinical pregnancy rate, n (%)	38.1 (437/1146)	41.6 (477/1146)	0.864 (0.731-1.022)	0.088
Twin pregnancy rate, n (%)	26.1 (114/437)	27.7 (132/477)	0.922 (0.688-1.236)	0.589
Miscarriage rate/pregnancy, n (%)	29.1 (127/437)	17.2 (82/477)	1.915 (1.378-2.660)	0.001
Early miscarriage rate, n (%)	21.1 (92/437)	17.2 (82/477)	1.285 (0.923-1.788)	0.137
Late miscarriage rate, n (%)	8 (35/437)	0 (0/477)	0.920 (0.895-0.946)	<0.001
Live birth rate, n (%)	26 (298/1146)	31.5 (361/1146)	0.764 (0.637-0.916)	0.004
Preterm birth rate, n (%)	20.1 (60/298)	16.6 (60/361)	1.265 (0.851-1.880)	0.245
<32weeks, n	7.72 (23/298)	1.66 (6/361)	4.948 (1.988-12.32)	<0.001
≥32weeks, n	12.42 (37/298)	14.86 (54/361)	0.806 (0.514-1.264)	0.346
The mean birth weight (g)	2913±743	3041±633		0.014
Low birth weight rate (%)	21.9 (76/347)	10.8 (48/444)	2.314 (1.562-3.427)	<0.001
Placenta praevia rate (%)	4 (12/298)	1.4 (5/361)	2.987 (1.040-8.578)	0.033
Preeclampsia rate (%)	6 (18/298)	2.8 (10/361)	2.256 (1.025-4.966)	0.038
Gestational diabetes rate (%)	15.1 (45/298)	13.8 (50/361)	1.106 (0.716-1.710)	0.649

After correcting for age and number of embryos transferred,multivariate analysis showed no statistically significant difference was found in the clinical pregnancy rate between the two groups (**Table 4**) (OR: 0.852, 95% CI: 0.717 to 1.013; P=0.070). After correcting for maternal age and singleton and twin pregnancy, the risk of low birth weight(OR:1.473, 95% CI:0.942 to 2.305; P=0.090), gestational diabetes(OR:1.166, 95% CI: 0.753 to 1.806; P=0.492) and preterm birth(OR:1.333, 95% CI:0.874 to 2.032; P=0.182) were not statistically significantly different between the two groups. However, in adenomyosis group, the rate of miscarriage(OR: 1.877, 95% CI: 1.351 to 2.608; P=0.000), placenta previa (OR: 2.996, 95% CI:1.040 to 8.632; P=0.042)and preeclampsia (OR: 2.287, 95% CI:1.030 to 5.080; P=0.042)were increased significantly, while live birth rate (OR: 0.541, 95% CI: 0.390 to 0.751;P=0.000)was reduced significantly than control group (**Table 4**).

**Table 4:** Comparison of clinical outcomes and obstetric complications between adenomyosis and control group.

	OR	95%CI	P value
Clinical pregnancy rate	0.852	0.717-1.013	0.070^#^
Miscarriage rate	1.877	1.351-2.608	0.000
Live birth rate	0.541	0.390-0.751	0.000
Low birth weight	1.473	0.942-2.305	0.090
Preterm birth	1.333	0.874-2.032	0.182
Placenta previa	2.996	1.040-8.632	0.042
Preeclampsia	2.287	1.030-5.080	0.042
Gestational diabetes	1.166	0.753-1.806	0.492

^#^Adjusted by maternal age, infertility type, No. of embryos transferred.

^#^Adjusted by maternal age, singleton and twin.

OR, odd ratio; CI, confidence interval.

## Discussion

Few and limited patient studies have examined reproductive outcomes in women with adenomyosis. Adenomyosis had a negative effect on IVF/ICSI outcomes in our large cohort study. Our study demonstrated that in adenomyosis patients treated with fresh embryo transfer, the live birth rate was significantly lower in women with adenomyosis than in the women with only tubal factor infertility (controls); patients with adenomyosis were also at an elevated risk of miscarriage, low birth weight and pregnancy complications.

In the aforementioned studies, we used TVS as the diagnostic tool for adenomyosis. The International Morphological Uterus Sonographic Assessment (MUSA) group reached an agreement in 2015 on terminology to use when describing myometrial lesions on 2D-TVS (17). The presence of two or more sonographic characteristics implies adenomyosis and can be used as a diagnostic tool (18). According to a recent article, seven factors should be considered when examining and describing a uterus with adenomyosis using ultrasound: presence, location, differentiation (focal/diffuse), appearance (cystic/non-cystic), uterine layer involvement, and extent and size of the lesion (19). However, there is still no universal international standard for ultrasound-based adenomyosis diagnosis.

The number of oocytes collected and available embryos were comparable in the patients with adenomyosis and the controls, and the clinical pregnancy rate was also comparable (38.1% in the women with adenomyosis versus 41.6% in the controls); therefore, adverse pregnancy outcomes due to the quality of the embryo were not considered. Miscarriage rate occurred in 29.1% of the women with adenomyosis and 17.2% of those without adenomyosis, which is consistent with the observations of previous studies (6, 20–23). The following are possible mechanisms for increased miscarriage risk in adenomyosis. First, disruptions at the endometrial–myometrium interaction influence endometrial features (24). Second, inflammatory responses and inordinate free radical formation can lead to abnormal implantation and miscarriage (25). Alterations in the inner myometrium in women with adenomyosis may be at the root of faulty reshaping of the myometrial spiral arteries from the initiation of decidualization, resulting in vascular resistance and an increased risk of defective deep placentation (24).

Some important reliability coefficients may differ between women with and without adenomyosis. This point may have been crucial in explaining discrepancies between available studies that have examined the impact of adenomyosis on IVF. Furthermore, coexisting gynaecological diseases such as endometriosis and uterine fibroids have been linked to poor pregnancy outcomes. Therefore, potential confounders in the study groups were well matched or excluded.

Pregnancy complications (such as hypertensive disorders of pregnancy and placenta previa) were significantly more common in the adenomyosis group than in the control group. This was consistent with previous reports (26). Therefore, the cause of this finding is that chronic inflammation caused by adenomyosis may impede deep placentation, contributing to the development of preeclampsia. These complications have been linked to impaired deep placentation caused by defective reshaping of spiral arteries in the endometrial junctional zone (22).

This study has several strengths, including a large sample size; strict inclusion and exclusion criteria to limit potential confounding bias; data analysis from the first IVF/ICSI cycle to overcome issues of lack of responsibility, biased assessment of outcomes, and predictive heterogeneity; and assessment of the clinically important outcome measure of live birth. Based on relatively large sample size, our data provide comprehensive insight into the adverse effect of adenomyosis on IVF-ET outcomes and obstetric complications, suggesting the importance of full consultation and active use of ART for women with adenomyosis. Furthermore, clinical and basic studies are called for more effective treatments to improve the outcomes. The observational essence of this study was hampered methodologically by the inability to completely control for selection and perplexing biases. However, the limitation of this study is that it is a retrospective cohort study, and further validation of the conclusions is needed in a prospective cohort study. Other restrictions include the diagnostic accuracy of non-invasive imaging technology for adenomyosis (the diagnosis should hopefully be conducted by histology) and the inability to exclude certain pathologies, such as peritoneal endometriosis.

In conclusion, our large, retrospective cohort study discovered a negative relationship between adenomyosis and fertility outcomes, particularly an increase in the miscarriage rate and obstetric complications associated with ART.

## Data Availability Statement

The raw data supporting the conclusions of this article will be made available by the authors, without undue reservation.

## Ethics Statement

This study was approved by The Peking University Third Hospital Ethical Review Committee approved the research (Approval no. LM2021243). The patients/participants provided their written informed consent to participate in this study.

## Author Contributions

All authors contributed to the article and approved the submitted version.

## Funding

The ‘Capital’s Funds for Health Improvement and Research (2014-1-4091)’ and ‘Natural Science Research General project of Shanxi Province(202103021224424)’ provided funding for this study as did the ‘Peking University Third Hospital Key Clinical Program (BYSY2015002)’.

## Conflict of Interest

The authors declare that the research was conducted in the absence of any commercial or financial relationships that could be construed as a potential conflict of interest.

## Publisher’s Note

All claims expressed in this article are solely those of the authors and do not necessarily represent those of their affiliated organizations, or those of the publisher, the editors and the reviewers. Any product that may be evaluated in this article, or claim that may be made by its manufacturer, is not guaranteed or endorsed by the publisher.
